# Post-auricular muscle reflex in the Middle Latency Evoked Auditory Response

**DOI:** 10.1016/S1808-8694(15)30499-7

**Published:** 2015-10-19

**Authors:** Carla Gentile Matas, Ivone Ferreira Neves, Flàvia Macarelli de Carvalho, Renata Aparecida Leite

**Affiliations:** 1 Communications Disorders, Professor of Speech and Hearing Therapy - FMUSP; 2 Sciences, Speech and Hearing Therapist - Speech and Hearing Therapy Program - FMUSP; 3 Speech and Hearing Therapist; 4 Sciences, PhD in Sciences - Graduate Program in Rehabilitation Sciences - Speech and Hearing Therapy Program - FMUSP

**Keywords:** hearing, auditory perception, auditory evoked potentials

## Abstract

The Middle Latency Auditory Evoked Response may be influenced by the post-auricular muscle reflex which occurs at the same latency of this potential.

**Aim:**

to evaluate the muscle reflex influence on the middle latency response, identifying the most appropriate place for response recording.

**Materials and Methods:**

Prospective study in which 40 normal hearing individuals, ranging in age from 18 to 40 years old, were assessed by Middle Latency Response with electrodes placed first on the mastoids, and then on the earlobes.

**Results:**

significant statistical differences were seen between the values found with electrodes placed on the mastoids and on the earlobes concerning the Na-Pa amplitude in C3/A1, C3/A2 and C4/A2, concerning the Na wave latency in C3/A2 and C4/A2, and concerning the Pa wave latency in C3/A1 and C3/A2. We found a higher occurrence of the post-auricular reflex when the electrode was placed on the mastoids, in all studied modalities.

**Conclusion:**

there was post-auricular muscle reflex interference upon the Middle Latency Response obtained when the electrodes were placed on the mastoid, and the most efficient electrode disposition in order to capture and to register more accurately this potential was on the earlobe.

## INTRODUCTION

The Middle Latency Evoked Auditory Potentials (MLEAP) were initially described in 1958^1^ as early responses with an initial latency of approximately 20 milliseconds (ms). Na, Pa, Nb and Pb waves are the most stable, which appear respectively around 18, 30, 40 and 50 ms after acoustic stimulus presentaion^2^. According to the literature, the Pa wave is usually the most robust MLEAP component^3^.

MLEAPs have multiple generators, such as the reticular formation and multisensorial divisions in the thalamus^3^, ^4^.

Besides assessing the dysfunctions which compromise the Central Nervous System auditory pathway, this potential can be utilized to establish electrophysiological thresholds in lower frequencies, assess cochlear implants, in perioperative monitoring^5^, ^6^ and to assess Auditory Processing Disorders (APD) ^7^.

The Central Auditory System evaluation, by means of recording middle latency evoked auditory potentials, requires placing the electrodes in pre-determined places. One example of electrode placement which allows for a good response recording and, consequently, a good diagnosis is associated to placing the electrodes in the C4 and C3 positions (right and left side temporoparietal junctions), Cz (vertex) and on the right and left side mastoids (M2 and M1) or right and left earlobes (A2 and A1) (10 – 20 International Electrode System) - C4 and C3 are considered active electrodes and M2, M1, A2 and A1 are considered reference electrodes. This electrode placement allows MLEAP to be registered ipsi and contralaterally to the stimulated ear (in many derivations), allowing for the comparison of wave latency and amplitudes between each hemisphere and the middle line.

Head and neck muscle activity impact on the recording of evoked potentials has been described in the literature, and such activity may cause doubts as to the neurogenic origin of these responses. This influence can be seen by means of the Vestibular Evoked Myogenic Potential - VEMP, which is captured with click or tone burst stimuli in intensities higher than 90 dBHL and reflects the activity of the vestibular system triggered by high intensity stimuli^8^.

A common difficulty found in recording MLEAP is that, when there are high levels of stimulation, many reflexes from scalp muscles happen in the same latency of evoked potentials, between seven and 50 ms^9^. These reflexes can influence MLEAP recording, causing doubts as to the neurogenic origin of these responses. Such reflexes, when present, influence the parameters of analyzed values such as wave latency and amplitudes, causing wrong values and, consequently, interfering in the audiological diagnosis being carried out, which should be quickly identified and avoided^9^.

Among these reflexes, the post auricular muscle reflex is the one most frequently observed. This happens between 14 and 19 ms after the acoustic stimulus presentation, it is usually recorded by the ipsilateral electrode to the sound and it is created similarly to the stapes muscle reflex, which afferent pathway is the VIII cranial nerve and the efferent is the VII cranial nerve. Having this reflex in this region is associated with muscle tension, thus depending on the patient's head position^10^.

The post-auricular reflex (PAR) activity is usually triggered by a sudden acoustic reflex of high intensity, which could be recorded by means of an electrode placed on the mastoid - and this is considered a myogenic response^11^.

It is likely that the anatomical structures involved in the post-auricular reflex are the cochlear nucleus, the superior olivary complex, the lateral lemniscus nucleus and very likely the inferior colliculus^11^.

Having the electrodes placed on the right and left mastoid to record the MLEAP, the presence of this post-auricular reflex can cause an alteration in wave latency and amplitude values - parameters used in the analysis of responses, interfering thus in the final diagnosis^11^.

Literature reports that the most efficient location of the electrode to minimize post-auricular reflex recording would be a non-encephalic region in the individual, such as the earlobes, for instance^10^. Although the post-auricular reflex interference is described in the MLEAP, there is still no routine for electrode placement on the earlobes, they are then preferable used on the mastoids.

Considering such aspects, we see that it is extremely important to study the MLEAP using different electrode locations.

Therefore, the present investigation aimed at assessing the interference of myogenic responses on the middle latency evoked auditory potential responses in audiologically normal individuals with different electrode locations (mastoids and earlobes), as well as comparing them among themselves.

## MATERIALS AND METHODS

This study was prospective, and approved by the Ethics Committee of our institution under protocol # 274/06. 20 male individuals and 20 females on the age range between 18 and 40 participated in this study, all with normal hearing.

For data collection we used the following equipment: otoscope (Heine Optotechnik, Herrsching Germany); middle ear analyzer (model GSI-33, Grason-Stadler, Inc., Milford, NH, USA); audiometer (model GSI-68, Grason-Stadler); supra-aural phone (model TDH-50, Telephonics Corp., Farmingdale, NY, USA); equipment for electrophysiological evaluation (model Traveler Express, Bio-logic Systems Corp., Mundelein, IL, USA).

Before the evaluations, the patient received information about the procedures which would be used for the study and, being in agreement to participate in it, they signed a free and informed consent form.

The individuals were initially submitted to an interview, in which data was collected about the presence of risk factors for hearing impairment, complaints of otitis, among other alterations associated with the external and middle ears.

Following that, we inspected the external acoustic meatus, aiming at seeing possible obstructions by cerumen. In the audiological evaluation we studied the acoustic immitance, tonal audiometry in the frequencies of 250, 500, 1000, 2000, 3000, 4000, 6000 and 8000 Hz, and vocal audiometry in order to select those individuals with normal hearing. Later on, we performed the BEAP with thin polarity click stimuli, presented to one ear only at 80 dB HL, at a presentation speed of 19.0 stimuli per second, with a total of 2,000 stimuli. The electrodes were positioned on the vertex (Cz) and the right and left mastoids (M1 and M2).

Considering the tonal audiometry, vocal audiometry, acoustic impedance and BEAP data, normal individuals were those who had:

• Tonal audiometry: threshold averages of the following frequencies: 500; 1,000 and 2,000 Hz lower than or equal to 25 dB HL^12^.
• Vocal audiometry: Speech Recognition Threshold (SRT) with responses equal to or up to 10 dB above the mean values of the auditory thresholds of the following frequencies: 500, 1,000 and 2,000 Hz in tonal audiometry^13^; Speech Recognition Thresholds (SRT) with correct answers between 90 and 100%, at the intensity of 30 dB above the SRT^14^.
• Acoustic immitance values: type A tympanometric curve^15^, ipsilateral acoustic reflexes present at the frequencies of 500, 1,000 and 2,000 Hz between 80 and 95 dB HL above air tonal threshold16 and collaterals present on the same frequencies aforementioned at intensity levels between 70 and 95 dB above the tonal threshold^17^, ^18^.
• BEAP: absolute latencies of waves I, III and V and interpeaks I-III, III-V and I-V within normal ranges, using values proposed by the Evoked Potential User Manual for the BIO-LOGIC equipment in individuals older than 24 months.

After individual selection, MLEAP recording started with the electrodes placed on the right and left mastoids, as well as on the left and right earlobes. In order to obtain this potential, the skin was cleaned with an abrasive paste, and later on the electrodes were fixed to the individual's skin by means of an electrolytic past and adhesive tape (micropore) in predetermined positions, according to IES 10-20 (International Electrode System).

The electrodes' impedance values were checked, and they had to be below 5 kOhms. The acoustic stimuli used to trigger the response was the thin polarity click, being monaurally presented at 70 dB HL, at a presentation speed of 1,000 clicks. The test was carried out in an electrically protected environment which was also acoustically isolated.

In the first MLEAP evaluation, the electrodes were positioned on the right and left mastoids (M2 and M1) (reference), right and left temporoparietal junctions (C4 and C3) (actives) and on the vertex (Cz).

In the second MLEAP evaluation, the electrodes were placed on the right and left earlobes (A2 and A1) (reference), right and left temporoparietal junctions (C4 and C3) (actives) and on the vertex (Cz).

**Figure 1:**
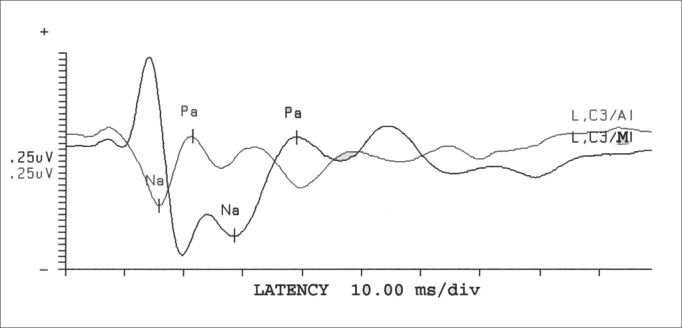
Presence and absence of myogenic response in face of sound stimulus in different electrode positions - L = left C3 = Left side temporoparietal junction A1 = Left earlobe M1 = Left mastoid

After recording MLEAP, the waves obtained were analyzed according to their amplitude and latency duration, as well as the presence or absence of post-auricular reflexes, for each assessment carried out (facing different electrode positioning) (Fig. 1). The presence or the absence of the post-auricular reflex was established taking into account wave morphology and latency duration.

At the end of this study, the data obtained with the electrodes fixed to both positions (1st and 2nd evaluations) was analyzed and compared.

MLEAP results were initially analyzed from mean values, medians and standard deviations of the latencies and amplitudes of waves Na, Pa and Nb, for each mode - ipsilateral (C3/A1 and C4/A2) and contralateral (C3/A2 and C4/A1), in the two assessment situation.

After this initial analysis, we compared the latency values of waves Na, Pa and Nb and Na-Pa and Pa-Nb amplitudes in the ipsilateral and contralateral modes between the two assessment situations (mastoid X earlobe), for that using the t Student test and a significance level of 5%.

Later on we analyzed the occurrence of post-auricular reflexes (presence and absence) in the four positions (C3/A1, C4/A1, C3/A2 and C4/A2), both with an electrode on the mastoid as well as on the earlobe, using the Wilcoxon Signed Rank Test with a significance level of 5% in order to check whether or not there was any difference in terms of the presence of these reflexes according to electrode positioning.

And finally, we analyzed this post-auricular reflex latency obtaining data related to the mean, median and standard deviation, on the four positions (C3/A1, C4/A1, C3/A2 and C4/A2), with the electrode placed on the mastoid and on the earlobe.

## RESULTS

Initially, we obtained the mean, median and standard deviation for the latency values for waves: Na, Pa, Nb and amplitudes NaPa and PaNb for both ears in the four positions (C3/A1, C4/A1, C3/A2 and C4/A2), with electrodes on the mastoids and on the earlobes. Afterwards, we did a comparative study of the means for each variable studied between the different positions of electrodes (mastoid X earlobe)

In comparing Na-Pa amplitude values obtained from mastoid and earlobe electrodes, we noticed a statistically significant difference for the following modes C3/A1, C3/A2 and C4/A2 (Table 1).

**Table 1:** Comparing Na-Pa amplitudes in MLEAP C3/A1, C4/A1, C3/A2, C4/A2 modes between mastoid and earlobe

Na-Pa Amplitude	C3/A1		C4/A1		C3/A2		C4/A2	
	MASTOID	EARLOBE	MASTOID	EARLOBE	MASTOID	EARLOBE	MASTOID	EARLOBE
Size	40	40	40	40	40	40	40	40
Mean	3,96	2,00	2,18	2,01	4,93	1,83	2,30	1,39
Median	1,58	1,32	1,37	1,31	1,94	1,33	1,76	1,16
Standard deviation	1,57	2,22	2,03	3,07	9,54	1,85	1,97	0,87
p-value	0,02		0,66		0,05		0,00	

C3 = Left side temporoparietal junction, C4 = Right side temporoparietal junction, A1 = Left ear, A2 = Right ear

We did not find statistically significant mean differences in comparing Pa-Nb amplitudes obtained from the electrodes on the mastoid and on the earlobe for any of the modes studied (Table 2).

**Table 2:** Comparing Pa-Nb amplitudes in MLEAP C3/A1, C4/A1, C3/A2, C4/A2 modes, between the mastoid and earlobe.

Pa-Nb Amplitude	C3/A1		C4/A1		C3/A2		C4/A2	
	MASTOID	EARLOBE	MASTOID	EARLOBE	MASTOID	EARLOBE	MASTOID	EARLOBE
Size	40	40	40	40	40	40	40	40
Mean	2,06	1,77	1,48	1,73	2,47	1,74	1,54	1,33
Median	1,46	1,28	1,27	1,17	1,45	1,18	1,29	1,06
Standard deviation	1,99	1,81	0,82	2,20	3,35	1,66	1,08	0,97
p-value	0,36		0,38		0,20		0,35	

C3 = Left side temporoparietal junction, C4 = Right side temporoparietal junction, A1 = Left ear, A2 = Right ear

Comparing the Na wave latency in mastoid and earlobe electrodes, we found a statistically significant difference for modes C3/A2 and C4/A2 (Table 3).

**Table 3:** Comparing the Na wave latencies in MLEAP C3/A1, C4/A1, C3/A2, C4/A2 modes between the mastoid and earlobe.

Na-Pa Amplitude	C3/A1		C4/A1		C3/A2		C4/A2	
	MASTOID	EARLOBE	MASTOID	EARLOBE	MASTOID	EARLOBE	MASTOID	EARLOBE
Size	40	40	40	40	40	40	40	40
Mean	19,39	18,66	18,78	18,62	19,24	18,49	19,56	18,23
Median	19,11	18,14	18,92	18,33	19,11	18,33	19,11	18,14
Standard deviation	2,49	3,31	2,74	4,13	2,09	2,96	2,45	3,00
p-value	0,22		0,84		0,15		0,01	

C3 = Left side temporoparietal junction, C4 = Right side temporoparietal junction, A1 = Left ear, A2 = Right ear

We found a statistically significant difference when we compared Pa wave latencies obtained from mastoid and earlobe electrodes for modes C3/A1 and C3/A2 (Table 4).

**Table 4:** Comparing Pa wave latencies in MLEAP C3/A1, C4/A1, C3/A2, C4/A2 modes between the mastoid and earlobe.

Na-Pa Amplitude	C3/A1		C4/A1		C3/A2		C4/A2	
	MASTOID	EARLOBE	MASTOID	EARLOBE	MASTOID	EARLOBE	MASTOID	EARLOBE
Size	40	40	40	40	40	40	40	40
Mean	34,12	32,01	34,07	32,87	34,94	32,61	33,18	32,50
Median	34,32	32,37	33,35	32,76	34,71	32,57	32,76	32,37
Standard deviation	4,64	5,75	4,72	4,61	3,89	5,55	5,14	4,74
p-value	0,08		0,33		0,04		0,33	

C3 = Left side temporoparietal junction, C4 = Right side temporoparietal junction, A1 = Left ear, A2 = Right ear

In comparing wave Nb latencies obtained from mastoid and earlobe electrodes, there was no statistically significant difference in any of the modes analyzed (Table 5).

**Table 5:** Comparing Nb wave latencies in MLEAP C3/A1, C4/A1, C3/A2, C4/A2 modes, between Mastoid and Earlobe.

Na-Pa Amplitude	C3/A1		C4/A1		C3/A2		C4/A2	
	MASTOID	EARLOBE	MASTOID	EARLOBE	MASTOID	EARLOBE	MASTOID	EARLOBE
Size	40	40	40	40	40	40	40	40
Mean	45,51	44,80	46,91	45,66	47,51	46,83	46,77	45,91
Median	45,44	43,68	45,63	44,27	46,41	44,07	45,44	44,07
Standard deviation	5,61	5,34	6,39	5,20	4,95	7,96	6,51	7,84
p-value	0,54		0,26		0,61		0,49	

C3 = Left side temporoparietal junction, C4 = Right side temporoparietal junction, A1 = Left ear, A2 = Right ear

Bellow, we show a study on the presence and absence of post-auricular reflex (PAR) on the four positions (C3/A1, C4/A1, C3/A2 and C4/A2), with electrodes on the mastoid and on the earlobe.

In studying the post-auricular reflex for the different electrode positions, we noticed a statistically significant difference for all the modes analyzed (Table 6).

**Table 6:** PAR occurrence distribution in MLEAP, both in the Mastoid and the Earlobe, for the C3/A1, C4/A1, C3/A2 e C4/A2 modes.

	C3/A1				C4/A1				C3/A2				C4/A2			
	MASTOID		EARLOBE		MASTOID		EARLOBE		MASTOID		EARLOBE		MASTOID		EARLOBE	
	Fr	%	Fr	%	Fr	%	Fr	%	Fr	%	Fr	%	Fr	%	Fr	%
present	12	30	0	0	8	20	0	0	12	30	0	0	8	20	1	2,50
absent	28	70	40	100	32	80	40	100	28	70	40	100	32	80	39	97,50
p-value	0,001				0,005				0,001				0,020			

C3 = Left side temporoparietal junction, C4 = Right side temporoparietal junction, A1 = Left ear, A2 = Right ear, Fr = Frequency, % = Percentage

After identifying the presence of a post-auricular reflex, we obtained the mean, median and standard deviation for the latency identifying the post-auricular reflex, we obtained the mean, median and standard deviation of the latency values for this reflex for both ears on the four positions (C3/A1, C4/A1, C3/A2 and C4/A2), with the electrode placed on the mastoid and earlobe; however, it was not possible to do a comparative statistical analysis of the mean values between the mastoid and earlobe because of the fact that only one ear had a post-auricular reflex with the electrode placed on the lobe (Table 7).

**Table 7:** PAR average values for both ears in the C3/A1, C4/A1, C3/A2 and C4/A2 modes, with the electrode placed on the mastoid and earlobe.

Nb latency	C3/A1		C4/A1		C3/A2		C4/A2	
	MASTOID	EARLOBE	MASTOID	EARLOBE	MASTOID	EARLOBE	MASTOID	EARLOBE
Size	12	0	8	0	12	0	8	1
Mean	14,24	—	14,28	—	14,37	—	14,38	14,43
Median	14,04	—	14,04	—	14,24	—	14,82	—
Standard deviation	0,95	—	0,83	—	0,85	—	1,65	—
p-value	—-		—-		—-		—-	

C3 = Left side temporoparietal junction, C4 = Right side temporoparietal junction, A1 = Left ear, A2 = Right ear

## DISCUSSION

In studying the mean latency values of waves Na, Pa, Nb and Na-Pa and Pa-Nb amplitude on the four modes (C3/A1, C4/A1, C3/A2 and C4/A2), both with the electrode placed on the mastoid as with it placed on the earlobe, it was observed that these values were higher when the electrodes were placed on the mastoid, except for the Pa-Nb amplitude on mode C4/A1, having that this value was higher with the electrode placed on the earlobe (Table 1, Table 2, Table 3, Table 4, Table 5).

Studying the specialized literature, we found some papers discussing the possibility of changing the latency and amplitude values of MLEAP waves considering different electrode positions, using the presence of PAR with electrodes placed on the mastoid^9^, ^19^.

Results show that in comparing the mean values of latencies and amplitudes of MLEAPs, statistically significant values were found in comparing Na-Pa amplitude values for modes C3/A1, C3/A2 and C4/A2 (Table 1), in comparing wave Na latency for modes C3/A2 and C4/A2 and comparing Pa wave latency for modes C3/A1 and C3/A2 (Table 3, Table 4 respectively).

Although literature reports the importance of MLEAP as a complementary exam to subjective tests10 and the existence of studies attempting to improve the objectiveness of the auditory function evaluation in patients with hearing loss^20^, considering the establishment of hearing thresholds regarding latency and amplitude values of its numerous deflections^20^, ^21^ we did not find in the literature studied many studies about the topic of this current paper, thus making it impossible to compare the findings previously discussed.

However, the data allows us to infer that the parameters which suffer the worst variation facing different electrode positions are the Na-Pa amplitude and Na and Pa wave latencies, which correspond to the first MLEAP waves, in other words, the Pa-Nb amplitude parameters (Table 2) and Nb wave latency (Table 5) are not affected by electrode position, having seen that these correspond to the later waves.

Such findings can be justified by the fact that the first waves are closer to the eventual PAR which can occur and be recorded with electrodes on the mastoid, interfering with the latency and amplitude values of the first MLEAP waves, and not on the latency and amplitude values of later waves.

In investigating the presence and absence of PAR in the four positions (C3/A1, C4/A1, C3/A2 and C4/A2), both in the mastoid electrode as well as that on the lobe, we noticed a greater occurrence of PAR with the electrode placed on the mastoid, on the four modes, when compared to the electrode placed on the earlobe (Tables 6).

Numerous studies in the literature show a greater likelihood of PAR occurrence and recording as one places the electrode on the mastoid, having seen that in this position we can record many reflexes coming from the scalp muscles, PAR among them^9^, ^11^. Moreover, the fact that we are using surface electrodes in electrophysiological tests makes it easier to capture such reflex, having seen that this electrode can be easily contaminated by artifacts of muscle activity or by external electrical interference^22^. According to the literature studied, one of the ways to prevent the recording of this reflex would be to place the electrodes on a non-cephalic region of the patient^10^.

In the present investigation we noticed that mean PAR latency values with the electrode placed on the mastoid were around 14 ms, in the four modes assessed(C3/A1, C4/A1, C3/A2 and C4/A2) (Table 7). These data are in agreement with those in the literature, which report the presence of this reflex between 14 and 19 ms after acoustic stimulus presentation, having the same latency of the MLEAP^10^.

## CONCLUSION

Comparing the data obtained with the variation in electrode positioning for MLEAP recording, we can see that when placed on the mastoids, we found higher values for Na and Pa waves latency and Na-Pa amplitude, as well as a greater PAR occurrence.

We can conclude that the presence of PAR changes latency and amplitude values for the first MLEAP waves, thus the importance of standardizing electrode positioning to record such potentials.

We recommend that when using MLEAP, the electrodes should be placed on the right and left earlobes, thus reducing PAR recording and, consequently, producing more reliable results.
